# Variable and Asymmetric Range of Enslaving: Fingers Can Act Independently over Small Range of Flexion

**DOI:** 10.1371/journal.pone.0168636

**Published:** 2016-12-19

**Authors:** Josien C. van den Noort, Nathalie van Beek, Thomas van der Kraan, DirkJan H. E. J. Veeger, Dick F. Stegeman, Peter H. Veltink, Huub Maas

**Affiliations:** 1 Biomedical Signals and Systems, MIRA Institute, University of Twente, Enschede, the Netherlands; 2 Department of Rehabilitation medicine, VU University medical center, MOVE Research Institute Amsterdam, the Netherlands; 3 Department of Human Movement Sciences, Faculty of Behavioural and Movement Sciences, Vrije Universiteit Amsterdam, MOVE Research Institute Amsterdam, The Netherlands; 4 Donders Institute, Department of Neurology and Clinical Neurophysiology, Radboud University Medical Centre, Nijmegen, the Netherlands; Georgia State University, UNITED STATES

## Abstract

The variability in the numerous tasks in which we use our hands is very large. However, independent movement control of individual fingers is limited. To assess the extent of finger independency during full-range finger flexion including all finger joints, we studied enslaving (movement in non-instructed fingers) and range of independent finger movement through the whole finger flexion trajectory in single and multi-finger movement tasks. Thirteen young healthy subjects performed single- and multi-finger movement tasks under two conditions: active flexion through the full range of movement with all fingers free to move and active flexion while the non-instructed finger(s) were restrained. Finger kinematics were measured using inertial sensors (PowerGlove), to assess enslaving and range of independent finger movement. Although all fingers showed enslaving movement to some extent, highest enslaving was found in adjacent fingers. Enslaving effects in ring and little finger were increased with movement of additional, non-adjacent fingers. The middle finger was the only finger affected by restriction in movement of non-instructed fingers. Each finger showed a range of independent movement before the non-instructed fingers started to move, which was largest for the index finger. The start of enslaving was asymmetrical for adjacent fingers. Little finger enslaving movement was affected by multi-finger movement. We conclude that no finger can move independently through the full range of finger flexion, although some degree of full independence is present for smaller movements. This range of independent movement is asymmetric and variable between fingers and between subjects. The presented results provide insight into the role of finger independency for different types of tasks and populations.

## Introduction

The number of different tasks in which we use our hands is very large. Examples are handwriting, grasping, typing, sport activities and playing musical instruments. Movements of the hand and fingers in these tasks need a complex control system. It has been shown that independent control of individual fingers in terms of movement and force is limited [1–5]. Together with voluntary single finger movement or force production, other fingers move or apply force as well. This dependency of movement and force of different fingers has been termed enslaving and has been attributed to both mechanical and neural factors [1–3, 5]. Mechanical factors include epimuscular myofascial force transmission (i.e., force transmission from muscle fibers onto their surrounding connective tissue network) [6–8] and mechanical coupling between the tendons of the muscles [1–3, 9, 10]. Neural factors include drive to motor units which innervate muscles fibers located in muscle heads associated with multiple fingers, spatial overlap of motor cortex areas for movements of different fingers and diverging central commands due to projections of single motor cortex neurons to several motor neurons in the spinal cord [2, 3, 10–15].

Previous studies have investigated finger independency for either force or movement tasks. These studies focused on involuntary force production or on finger movement in the non-instructed fingers. In terms of force, it has been shown that enslaving is present for each finger and is largest for the neighboring finger [2, 16]. The cause of the enslaving effect in force tasks is thought to be primarily neural, because in these static tasks relatively minimal movement occurs.

If the fingers are moved, the structures providing the mechanical coupling between tendons and muscles may experience more strain, in particular after a certain range of movement is achieved. Hence, also mechanical factors may play a role during finger movements [1]. During fast finger flexion, a time delay between movement of the non-instructed finger with respect to the instructed finger has been reported [17]. This suggests that there is a range of independent movement of an instructed finger before non-instructed fingers start to move. Therefore, we hypothesize that for small movements finger independency is high. Not many studies have looked into this range of movement, especially not during natural, multiple finger movement.

Movements of all three joints of a finger as well as movements of multiple fingers simultaneously, better resemble movements in daily life. However, movement enslaving has mainly been reported for a single joint or single-finger tasks [1, 16, 17], whereas only one study investigated whole finger movements including all three finger joints (metacarphophalangeal joint (MCP), proximal and distal interphalangeal joints (PIP and DIP) [4]. Outcomes for enslaving could be different depending on the included number of joints, as this will affect amount of length changes of the muscle-tendon units [18, 19]. Furthermore, constraining some of the joints does influence the finger movement, whereas study of all finger joints that are free to move and fully flex does better represent natural finger movements.

Most previous studies limited the expression of the extent of finger independence to a single outcome parameter based on the total range of motion (ROM) (enslaving effect or individuation index). An evaluation of the whole trajectory of finger movement might give further insights into finger independency, especially in a range of independent movement of an instructed finger (i.e., before the non-instructed fingers start to move). This could be very relevant in for example musicians who predominantly use small finger movements [20].

Therefore, the overall aim of the present paper is to assess the extent of finger independency during full-range finger flexion, including all finger joints. In particular this will be done by the study of (i) finger enslaving and (ii) range of independent finger movement of instructed fingers through the whole finger flexion trajectory in single and multi-finger movement tasks.

## Methods

### Subjects

Thirteen young healthy subjects participated in the study (age 22–30 years of age, 7 males, 6 females). Exclusion criteria were any known neuromuscular disorder, disability in the upper limb or surgery in the last two years, or experience with playing musical instruments with repetitive individual finger movements for more than two years over the course of the past five years (the latter because of a high degree of finger independency due to training or the risk of signs of focal task specific dystonia [21–23]). The medical ethical board of the Radboud University Nijmegen approved the study protocol. All subjects signed an informed consent.

### Equipment

**Fig 1:**
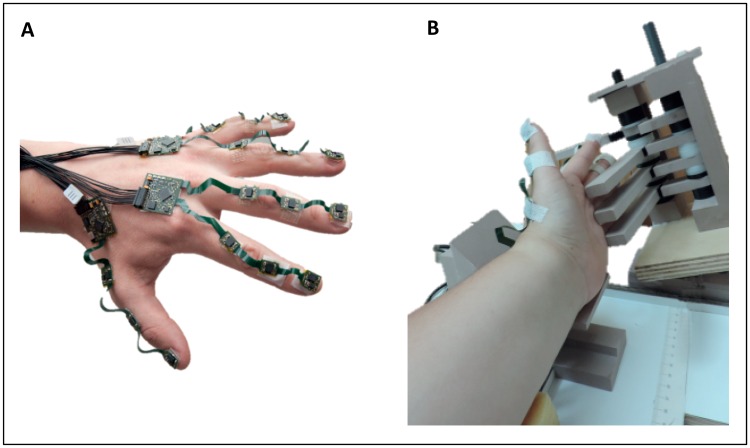
**(A)** Measurement set-up showing the PowerGlove with 18 sensor units on the fingers and hand (Kortier et al. 2014 [24], van den Noort et al. 2016 [25]). The sensors were attached to the dorsal side of the left hand (3 sensors), on the metacarpal, proximal and distal phalanges of the thumb (3 sensors) and on the proximal, middle and distal phalanges of the index, middle, ring and little fingers (12 sensors) using small Velcro straps. The arm was placed on a custom-made arm-rest during the measurements. **(B)** A system with small wooden bars provided restriction of the non-instructed fingers, as part of the arm-rest.

To measure finger movements, a recently developed measurement instrument which assesses 3D hand and finger kinematics was used (the PowerGlove [24, 25]). The PowerGlove consists of multiple miniature inertial and magnetic sensors (IMMS, containing 3D accelerometers, gyroscopes and magnetometers) that can be placed on each finger segment (Fig 1A). Eighteen PowerGlove sensor units [24, 25] were attached to the dorsal side of the left hand, on the metacarpal, proximal and distal phalanges of the thumb and the proximal, middle and distal phalanges of the index (i), middle (m), ring (r) and little (l) fingers using small Velcro straps. Prior to measurement, an anatomical calibration procedure (sensor to segment calibration) was performed to determine the sensor-to-segment coordinate systems of the PowerGlove as described in [24, 25].

### Protocol

The subjects were seated in a chair while the arm was placed on a custom-made arm-rest with a palmar position of the hand of 45° (Fig 1B). Subjects were asked to perform various finger movements through the full range of motion (ROM) until the tip(s) of the finger(s) touched the palm of the hand (Table 1). Two different conditions were performed: (1) voluntary active flexion with all fingers free to move (ACT), and (2) active flexion where the non-instructed finger(s) were restrained in a fully extended position (RES) (Fig 1B). Subjects were asked neither to pay attention to the non-instructed fingers, nor to resist any movement in these fingers, hence, to allow involuntary finger movement in a natural way. The initial position of the hand prior to the movement was a zero degrees joint angle in all finger joints, i.e. no flexion or extension. The velocity of the flexion movements was paced with a metronome (0.5 Hz). For each task, including single- and multi-finger movements (Table 1), five repetitions were performed. Kinematic data from the PowerGlove were recorded with a sample frequency of 100Hz.

**Table 1:** Flexion finger tasks: subjects were asked to perform various finger movements through the full range of motion (ROM) until the tip(s) of the finger(s) touched the palm of the hand.

Tasks		
Single-finger tasks	Two-fingers tasks	Three-fingers tasks
1. Index (i)	5. Index and middle (im)	8. Index, middle and ring (imr)
2. Middle (m)	6. Middle and ring (mr)	9. Middle, ring and little (mrl)
3. Ring (r)	7. Ring and little (rl)	
4. Little (l)		

### Data analysis

To obtain joint orientations from movement trials, the data of the PowerGlove were processed using a custom-made, Matlab-based algorithm applying the anatomical segment calibration and information from gyroscopes, accelerometers and magnetometers by using an extended Kalman filter algorithm that fuses all sensor inputs and a biomechanical hand model [24].

For both ACT and RES conditions, finger flexion movements of the middle three of the five repetitions were analyzed. Mean joint angles over these repetitions were calculated for the MCP, PIP and DIP joints of all four fingers separately, over each time point in the time-normalized flexion movement. Subsequently, the sum of the MCP, PIP and DIP joint angles was calculated per finger as an estimate of whole finger movement through the flexion movement. For the total range of motion of the flexion movement, the maximal angle was calculated of this sum angle, which is further named the ΣROM.

For comparison with the literature, both enslaving effect and individuation index were calculated per finger. To assess enslaving in the ACT condition, the enslaving effect for each of the non-instructed fingers (based on [2]) was calculated for all ACT movement tasks (single- and multi-finger tasks as listed in Table 1) by calculating ΣROM of the non-instructed finger relative to the ΣROM of the instructed finger (in percentage):

$${\text{enslaving effect }}_{\text{non-instructed finger}}=\frac{{\left(\Sigma \text{ROM}\right)}_{\text{non-instructed finger}}}{{\left(\Sigma \text{ROM}\right)}_{\text{instructed finger}}}\cdot 100\%$$ (1)

For multi-finger movement tasks with multiple instructed fingers, the ΣROM of the instructed finger adjacent to the non-instructed finger of interest was used.

Furthermore, the individuation index of an instructed finger (based on [1]) was calculated for the single-finger ACT movement tasks. This was done using the ΣROM of the instructed finger relative to the mean ΣROM of all three non-instructed fingers (value between 0 and 1):

$${\text{individuation index}}_{\text{instructed finger}}=1-\left(\frac{{\left(\text{mean }\Sigma \text{ROM}\right)}_{\text{non-instructed fingers}}}{{\left(\Sigma \text{ROM}\right)}_{\text{instructed finger}}}\right)$$ (2)

In addition to outcomes in literature, new measures have been used to assess independency of instructed fingers through the full-range finger flexion trajectory. First, a *δ* individuation index was calculated per instructed finger for the single-finger ACT movement tasks. For this, we used delta values (*δ*), i.e. the change in joint angle of the instructed finger (in steps of 10% ΣROM) relative to the change in mean joint angle of the three non-instructed fingers (value between 0 and 1). In this case, joint angle is referring to the sum of MCP, PIP and DIP joint angles over the movement trajectory.

$$\delta {\text{ individuation index}}_{\text{instructed finger}}=1-\left(\frac{{\left(\delta \text{ mean joint angle}\right)}_{\text{non-instructed fingers}}}{{\left(\delta \text{ joint angle}\right)}_{\text{instructed finger}}}\right)$$ (3)

**Fig 2:**
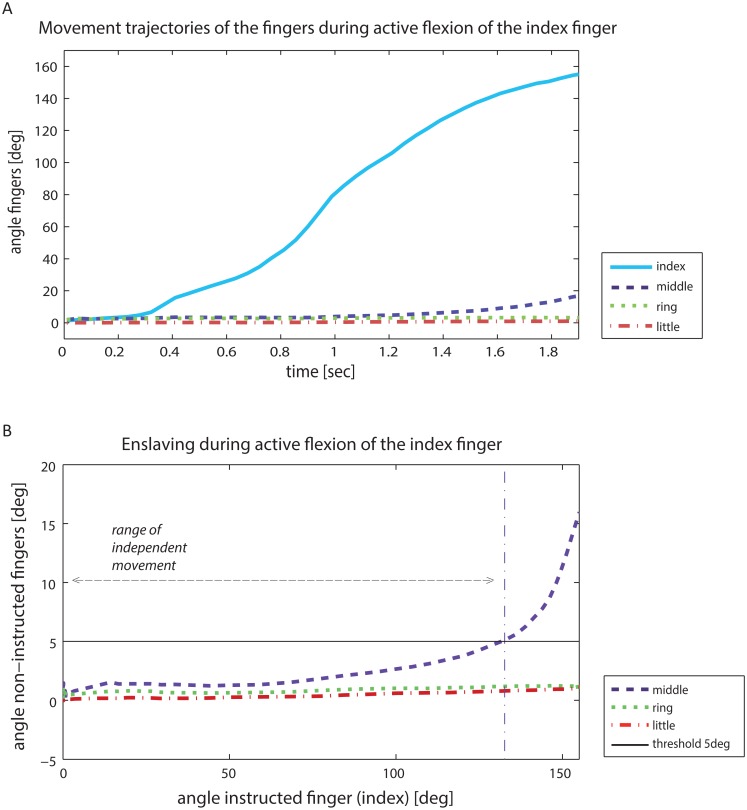
**(A)** Typical example of the movement trajectory (angle, y-axis) in time (x-axis) of the four fingers during active flexion of an instructed finger (index). **(B)** Corresponding typical example of enslaving in non-instructed fingers (y-axis) during active flexion of the instructed index finger (x-axis). The range of motion angle of a finger was calculated as the sum of the MCP, PIP and DIP joint angle excursions. Enslaving threshold was set at a change of more than 5 degrees of motion (black solid line) in the non-instructed finger. Based on this threshold, the range of independent movement of the instructed finger was defined, shown with the vertical thin dotted-dashed lines corresponding to the movement threshold of the non-instructed finger(s).

Furthermore, the range of independent movement of the instructed finger with respect to its ΣROM (i.e., at what percentage of ΣROM of the instructed finger did the non-instructed finger(s) start to move) was determined for all tasks in the ACT condition. The enslaving threshold (start of non-instructed finger movement) was defined as a change in joint angle of more than 5 degrees (Fig 2), based on reported thresholds to detect finger movements [26, 27].

### Statistical analysis

Paired-sampled T-tests were performed to compare ΣROM in ACT versus RES conditions of the instructed finger in the single-finger tasks. Furthermore, repeated measures ANOVA with post-hoc pairwise comparisons were performed to test significant differences within and between single- and multi-finger tasks in the ΣROM and enslaving effects of the non-instructed fingers, in the individuation index and the *δ* individuation index of the instructed fingers (only single-finger tasks), and in the range of independent movement of the instructed fingers. Prior to analyses, a Shapiro—Wilk test was used to test whether the data were normally distributed. When data were not normally distributed, the related samples Wilcoxon signed rank test was used to test for the significance of differences. A p-value of <0.05 was considered to indicate significance.

## Results

### Finger enslaving

During active flexion, all non-instructed fingers showed some degree of movement. The ΣROMs (the total range of motion of the flexion movement, as the sum of the maximal MCP, PIP and DIP joint angles per finger) during both single- and multi-finger tasks in all fingers are presented in Table 2 (see also S1 File). For the non-instructed fingers, the highest ΣROM was observed in the fingers adjacent to the instructed finger. Standard deviations between subjects were high, especially in the multi-finger tasks.

**Table 2:** The ΣROM (in degrees) during all active finger flexion tasks averaged over all 13 subjects.

	*index*	*middle*	*ring*	*little*
**Single-finger tasks**				
*i*	**155±27**	32±15 *	11±9	6±5
*m*	41±12	**166±30**	68±31 *^	16±8
*r*	20±35	52±36 *^	**151±32**	42±25
*l*	10±7	22±18	70±55 *	**131±40**
**Two-fingers tasks**				
*im*	**160±31**	**166±34**	86±40 *^	30±25
*mr*	47±17	**186±36**	**163±35**	66±24 *
*rl*	10±11	77±29 *^	**149±46**	**138±51**
**Three-finger tasks**				
*imr*	**171±35**	**182±39**	**158±36**	74±26
*mrl*	50±32	**170±48**	**167±52**	**136±41**

Values are mean±standard deviation in degrees of ΣROM

**bold** values = ΣROM instructed finger(s)

* = significantly highest ΣROM (p<0.05) in non-instructed finger compared to other non-instructed fingers in a particular finger flexion task

^ = non-parametric testing

i = index; m = middle; r = ring; l = little

ΣROM = sum of ROM of MCP, PIP and DIP joint angle per finger

ROM = Range of Motion, i.e. maximal angle of a joint during finger flexion

MCP = metacarphophalangeal joint; PIP = proximal interphalangeal joint; DIP = distal interphalangeal joint

The enslaving effects of the non-instructed fingers, calculated as ΣROM of the non-instructed finger relative to ΣROM of the (adjacent) instructed finger (in percentage), are presented in Table 3. In line with ΣROM, the highest enslaving effect was found in the adjacent non-instructed finger, whereas non-adjacent fingers showed lower degrees of enslaving. Of all fingers, the ring-finger showed the significantly highest enslaving effect, particularly in the m-, im- and l-tasks (40.3–64.0, p<0.05). This indicates a large influence of instructed middle and little finger on the enslaving ΣROM of the non-instructed ring finger. Also the middle finger showed a significant high enslaving effect in the rl-task (56.0). The index finger showed the significantly lowest enslaving effect (7.6–28.8), indicating low influence of movement in (adjacent) instructed fingers on the ΣROM of the non-instructed index finger.

**Table 3:** The enslaving effect of the non-instructed fingers over all 13 subjects (in % of ΣROM adjacent instructed finger).

	*index*	*middle*	*ring*	*little*
**Single-finger tasks**				
*i*		21.3±8.8 *	6.9±5.2	3.4±3.1
*m*	24.9±10.7		40.3±14.6 *	10.2±4.8
*r*	12.6±21.0	37.5±29.6 *^		30.8±21.3
*l*	8.6±4.6	24.6±14.4	64.0±28.5 *	
**Two-fingers tasks**				
*im*			51.9±23.7 *^	18.1±15.4
*mr*	24.6±7.0			37.4±13.6 *
*rl*	7.6±6.5	56.0±23.5 *		
**Three-finger tasks**				
*imr*				49.0±19.1
*mrl*	28.8±13.0			

Values are mean±standard deviation

Enslaving effect is calculated as ΣROM of the non-instructed finger relative to ΣROM of the (adjacent) instructed finger (in percentage)

* = significantly highest enslaving effect (p<0.05) in non-instructed finger compared to other non-instructed fingers in a particular finger flexion task

^ = non-parametric testing

i = index; m = middle; r = ring; l = little

ΣROM = sum of ROM of MCP, PIP and DIP joint angle per finger

ROM = Range of Motion, i.e. maximal angle of a joint during finger flexion

MCP = metacarphophalangeal joint; PIP = proximal interphalangeal joint; DIP = distal interphalangeal joint

Adjacent fingers showed a mutual and symmetrical enslaving effect on each other during the single-finger tasks (Table 4, 2^nd^ column). For example, the enslaving effect of the instructed index finger on the enslaving of the adjacent non-instructed middle finger (21.3, see Table 3) was not significantly different to the enslaving effect of the instructed middle finger on the enslaving of the adjacent non-instructed index finger (24.9, see Table 3).

**Table 4:** Differences and (a)symmetry in enslaving effect of non-instructed finger(s) and in range of independent movement of instructed finger(s) in single- and multi- finger flexion tasks.

Non-instructed (instructed) finger comparison	Enslaving effect non-instructed finger		Range of independent movement instructed finger	
	mean difference±SD	significance	mean difference±SD	significance
**Single-finger task comparisons**				
m(i)–i(m)	-4±11%	p = 0.314 ^	31±28%	p = 0.011 *^
m(i)–m(r)	-29±33%	p = 0.260 ^	32±34%	p = 0.028 *^
r(m)–m(r)	3±22%	p = 0.196 ^	-8±20%	p = 0.507 ^
r(m)–r(l)	-11±22%	p = 0.345 ^	-23±34%	p = 0.093 ^
l(r)–r(l)	-33±31%	p = 0.146	-12±31%	p = 0.575 ^
**Multi- vs. single-finger tasks**				
i(mrl)–i(mr)–i(m)		p = 0.577		p = 0.373
i(mr)–i(m)	-0.3±10%	p = 0.314 ^	7±27%	p = 0.959 ^
i(mrl)–i(mr)	4±15%	p = 0.416	5±17%	p = 0.463 ^
i(mrl)–i(m)	4±20%	p = 0.917 ^	15±31%	p = 0.310 ^
m(rl)–m(r)	19±26%	p = 0.059 ^	-6±10%	p = 0.071
r(im)–r(m)	12±14%	p = 0.001 *^	20±41%	p = 0.807 ^
l(imr)–l(mr)–l(r)		p = 0.081		p = 0.053
l(mr)–l(r)	7±14%	p = 0.610	-15±20%	p = 0.017 *^
l(imr)–l(mr)	11±13%	p = 0.035 *	25±39%	p = 0.059 ^
l(imr)–l(r)	18±15%	p = 0.033 *	8±32%	p = 0.721 ^

* = p<0.05

^ = non-parametric testing

i = index; m = middle; r = ring; l = little

Negative values (-) means: parameter in first finger(task) combination is lower than in second finger(task) combination

SD = standard deviation

Moving multiple fingers did change the ring and little finger enslaving. The non-instructed ring finger showed an increased enslaving during multi-finger movement (12±14% (mean ±standard deviation), p = 0.001), indicating an additional effect of the index finger on the ring finger (Table 4, 2^nd^ column). Furthermore, the enslaving effect in the non-instructed little finger significantly increased during three-finger task movements compared to two- and single-finger movement (l(imr) > l(mr): 11±13%, p = 0.033; l(imr) > l(r): 18±15%; p = 0.035). This indicates that movements in the index and middle finger have effect on the little finger movement. No changes due to movement of multiple fingers were found in both the index finger enslaving and the middle finger enslaving.

**Fig 3:**
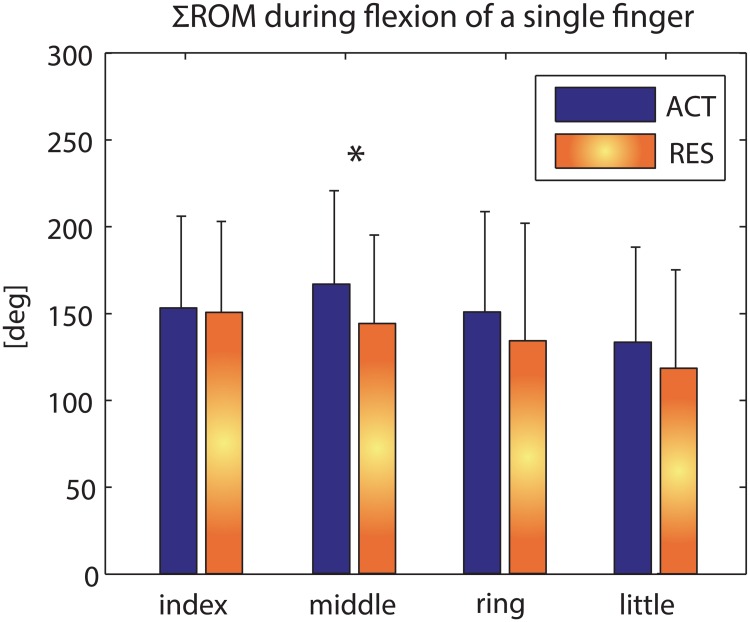
ΣROM (mean and standard deviation) of the fingers during single-finger movement tasks (n = 13, ACT = active flexion (blue bar), RES = flexion with restriction in non-instructed fingers (orange bar)). The asterisk indicates a significant difference in ACT versus RES.

Restriction of non-instructed fingers in single-finger flexion tasks (RES condition) did affect the ΣROM of the instructed middle finger. The ΣROM for the middle finger was lower when the non-instructed fingers were restricted compared to the same task without constraints (ACT condition) (23±29°, p = 0.015). For other fingers, no differences between conditions were found (Fig 3).

### Finger range of independency

**Fig 4:**
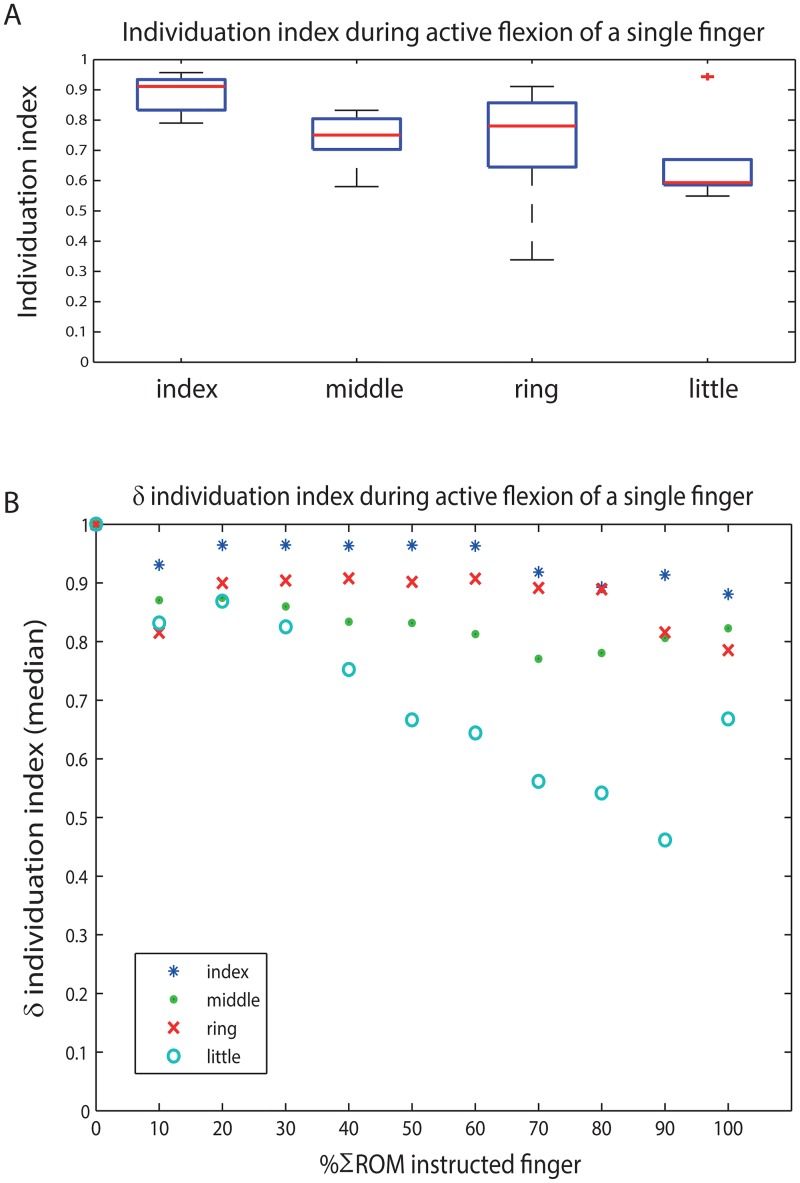
**(A)** Individuation indices of each instructed finger during single finger movement tasks in 13 young healthy subjects. The closer the value is to 1, the more independent a finger could be moved. The boxplots show the median (red line), 1st and 3rd quartiles (blue box), smallest and largest values (whisker with black lines) and outliers (red cross, >1.5 interquartile range) over the 13 subjects. The significantly highest individuation index, indicating the highest level of independence compared to the other fingers, was found for the index finger. **(B)** *δ* individuation indices of each instructed finger over the movement trajectory, per 10% ΣROM. At 70% ΣROM, the *δ* individuation index of the index finger decreased significantly compared to values below 70% ΣROM. From 60%-80% ΣROM, the *δ* individuation index of the ring finger was significantly lower to values outside that range. Also, the index finger had highest *δ* individuation indices compared to the little finger from 10% onwards, and to the middle finger from 20% onwards. At 40% and 60% ΣROM the middle finger showed a lower *δ* individuation index compared to the ring finger.

The significantly highest individuation index, indicating the highest level of independence compared to the other fingers, was found for the index finger (Fig 4A). Over the movement trajectory, the *δ* individuation index of the index finger at 70% ΣROM decreased significantly compared to values below 70% ΣROM (Fig 4B) (see also S1 File). From 60%-80% ΣROM, the *δ* individuation index of the ring finger was significantly lower to values outside that range. Furthermore, the index finger had significantly higher *δ* individuation indices compared to the little finger from 10% onwards, and to the middle finger from 20% onwards. At 40% and 60% ΣROM the middle finger showed a significantly lower *δ* individuation index compared to the ring finger.

**Fig 5:**
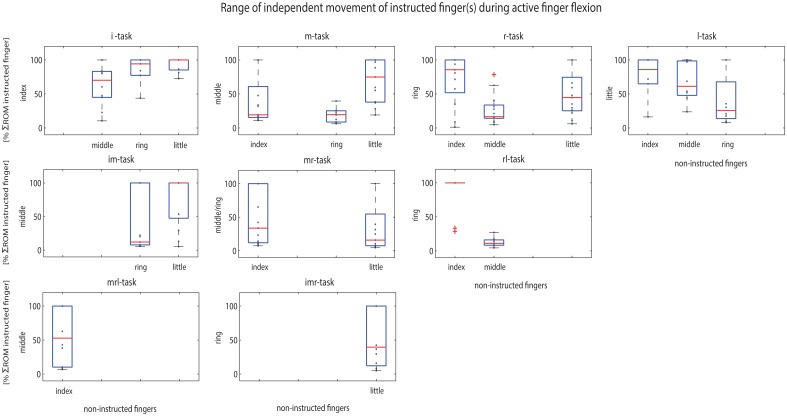
Boxplots showing the range of independent movement of the instructed finger(s) of all 13 subjects. This was defined as follows: where in the ΣROM (%) of the instructed finger(s) (vertical axes) the non-instructed finger(s) start(s) to move (horizontal axes). Data is presented for all finger movement tasks (single and multi; i = index, m = middle, r = ring, l = little). For multi-finger movement tasks, the used ΣROM of one of the adjacent instructed fingers is used. Individual results (mean over trials) per subject are presented in the blue dots. The boxplots show the median (red line), 1^st^ and 3^rd^ quartiles (blue box), smallest and largest values (whisker with black lines) and outliers (red cross, >1.5 interquartile range). Start of enslaving is defined as a change of more than 5deg finger movement (ΣROM) of the non-instructed finger(s) [26, 27]. A value (on the vertical axis) close to zero means enslaving early in the ΣROM of the instructed finger, whereas a high value means late or no enslaving effect, i.e. near or at the end of the ΣROM of the instructed finger. If movement of the non-instructed finger was less than 5deg, i.e. no enslaving effect, the enslaving value has been presented as being at the end ΣROM (100%) of the instructed finger because of visualisation purposes. In single finger movement tasks, it can be seen that the adjacent non-instructed finger starts to move first before the other non-instructed finger(s) start(s) to move.

Prior to the occurrence of movements in the non-instructed fingers, we observed that each of the instructed fingers could be moved independently for some range (on average between 13% and 61% of its ΣROM). This range was variable between fingers, subjects and tasks (Fig 5) (see also S1 File) and significantly highest for the index finger (i-task) compared to other tasks and fingers (61±29%). At group level, the index and middle fingers showed asymmetry in the range of independent movement of the instructed finger, i.e. the instructed middle finger showed a smaller range of independent movement (31±28%, p = 0.011) than the index finger. Furthermore, movement of the non-instructed middle finger started after a smaller range of instructed finger movement during ring finger flexion than during index finger flexion (32±34%, p = 0.028) (Table 4, 3^rd^ column). For the other single-finger tasks, no differences were found.

The start of enslaving movement in the little finger was affected by multi-finger movement. Movement of the middle finger in addition to movement of the ring finger caused a decrease in range of independent movement, i.e. the non-instructed little finger started to move at a smaller ring finger angle (15±20%, p = 0.017) (Table 4, 3^rd^ column). No effects were found of multi-finger movements on the range of independent movement of the index, middle and ring finger (Table 4, 3^rd^ column).

## Discussion

In this paper we aimed to assess the extent of finger independency during full-range finger flexion by the study of finger enslaving of non-instructed fingers and range of independent finger movement of instructed fingers through the whole finger flexion trajectory. Overall, as hypothesized, our results indicated that while no finger can move independently through the full-range of finger flexion, some degree of full independence is present for smaller movements (on average 13–61% of the ΣROM).

### Finger enslaving

In line with previous studies [2, 16, 17], we found the highest enslaving effects in the adjacent fingers. Almost all non-instructed fingers showed enslaving to a certain degree, which confirms that no finger can move independently through the full range of motion.

We found high enslaving effects in particular in the middle and ring finger. Other studies also showed high enslaving of these fingers [4, 17]. In our study, the influence of little finger movement on the enslaving of the ring finger was highest, whereas high enslaving effects were also found in the middle finger during ring and little finger flexion. In addition, the middle finger was the only finger affected by restriction in movement of non-instructed fingers.

The index finger was the most independent finger, as reflected by the lowest enslaving effect and the highest individuation indices. This has also been shown previously by others for both movement [4, 16, 17] and force tasks [16]. Lang and Schieber found the highest individuation index for the little finger whereas our results showed lowest individuation index for this finger [1]. Others reported lowest individuation index for the ring finger [4, 17].

Mutual enslaving effects of adjacent fingers were found to be symmetrical during single-finger tasks. This is in line with previously reported results in both movement [17] and force tasks [28]. Our results from the multi-finger tasks showed that the addition of instructed movement by non-adjacent fingers changes the extent of the enslaving effect. Little finger enslaving during ring finger movement was increased by movement in the index and middle fingers. Ring finger enslaving during middle finger movement was increased by movement in the index finger. Effect of non-adjacent fingers on enslaving has been reported previously by [17]. They showed that the little finger caused enslaving movement in the middle finger. They did not find, however, a reciprocal effect, i.e. an effect of middle finger on little finger. Their study was focussed to the DIP joint and single-finger tasks and, therefore, did not provide insight into additional enslaving effects in multi-finger tasks.

Differences between our results and those of other studies can be explained by the number of joints that are involved in the movement, and/or by the range of movement. Lang and Schieber instructed to move the MCP joint while the PIP and DIP joint were fixed in full extension [1]. Li et al. studied the isolated movement of the DIP joint while the MCP and PIP joints were restricted [17]. Each extrinsic muscle (flexor digitorum profundus (FDP) and flexor digitorum superficialis (FDS)) has a specific moment arm around a specific joint axis [18]. The FDP is the only muscle that inserts at the DIP joint, whereas both FDP and FDS are acting at the PIP and MCP joints [29]. Movement of multiple joints in comparison to movement of one joint does, therefore, increase the extent of muscle-tendon unit length changes of the extrinsic muscles [18, 19]. The extent of enslaving may be dependent on the number of finger joints involved. In tasks in daily life, often several joints and fingers are involved. Therefore, the study of finger movement with all three finger joints free to move is of relevance for interpretation of the role of finger enslaving in daily practice.

Furthermore, differences in finger range of movement may also explain differences between studies. For example, index and middle finger enslaving movement during active flexion of the little finger (l-task) started only over 50% of the ΣROM of the little finger (Fig 5). The ΣROM of the little finger was found to be 138 degrees (Table 2). This means that if a range of 69 degrees of little finger flexion is not reached, the index and middle finger will not show any movement and, subsequently, the individuation index of the little finger will be higher. This can explain that the small-arc movements (about 40 degrees MCP flexion) in the study by Lang and Schieber (2004) resulted in higher individuation indices for the middle, ring and little finger [1], compared to large-arc movements (about 75 degrees MCP flexion). Direct comparison with other studies for ΣROM is difficult since these are not clearly reported or only one joint was included.

### Range of independent movement

Although we have shown that fingers cannot move independently, each finger shows a range in which independent movement does occur. The index finger had the highest *δ* individuation index over the movement trajectory, which only significantly decreased at 70% ΣROM and onwards. Furthermore, the index finger showed the highest range of independent movement (on average 61%). As mentioned before, a similar measure as the range of independent movement has only been previously investigated for the DIP joint, expressed as a time delay between movement in instructed and non-instructed fingers [17]. The time delay for the index finger was reported to be highest confirming the highest range of independent movement.

This independent movement range has not been studied previously including all finger joints, neither with regard to symmetry between adjacent fingers nor concerning the effects of non-adjacent fingers in multi-finger tasks. Different from the enslaving effect, which only takes into account the total ΣROM, the range of independent movement analysis in this study showed that for the index, middle and ring finger, the effects on each other during the movement trajectory is not symmetrical. Multi-finger tasks revealed also the effects of non-adjacent fingers. Adding middle finger flexion to ring finger flexion resulted in an earlier start of enslaving movement in the little finger although movement of the little finger in addition to the ring finger did not change the start of the middle finger enslaving movement.

Our finding that a range of independent movement exists, seems in agreement with the presence of mechanical coupling [5, 6, 8, 12]. We propose that mechanical connections between the muscle heads or tendons corresponding to each of the non-instructed fingers are initially slack and need a certain relative displacement to produce high enough forces to causes finger movement.

The range of independent movement is asymmetrical, and variable between adjacent fingers (index, middle, ring) and between subjects. Characteristics of inter-tendinous connections might therefore be direction dependent. Furthermore, a large variability in muscle and tendon connections exists between subjects [30, 31] and might explain the variability in our results. The middle finger did show asymmetry towards the index versus the ring finger. Tendon connections between middle and ring finger might therefore be less slack than connections between middle and index finger, or, alternatively, neural factors might play a larger role for the ring finger than for the index finger [1].

Neural factors, such as motor units innervating multiple muscles of different fingers, spatial overlap in the motor cortex hand area and diverging central commands [2, 3, 10, 11, 28, 32] have been indicated to cause force enslaving. Li et al. [17] suggested that neural factors could also result in a range of independent movement, by differences in reflex pathways or differences in the cortical connections in the primary motor cortex of the hand. A delay of movement of the non-instructed fingers may be the results of excitation via gamma driven reflexes, instead of direct excitation by the alpha motor neurons [17]. Alternatively, Li et al. suggested that finger specific cortical cells might be excited first for the instructed finger and through horizontal cortical connections followed by those for non-instructed fingers, also causing a delay [17]. While most neurones in the motor cortex in monkeys are shown to be active during movements of multiple fingers, in single-finger movement activity of neurones is distributed over the primary motor cortex [11, 33]. Transcranial Magnetic Stimulation applied over the motor cortex in humans revealed little interaction among fingers in force-tasks [34]. Such time delays in excitation [17] might be determined by performing finger flexions at different movement velocities, in which the time delay then is hypothesized to be constant, and by evaluation of muscle activity in the muscle bellies of the FDP and FDS, using electromyography (EMG).

However, our preliminary data suggest no such timing in muscle excitation [35]. Furthermore, in contrast to enslaving movement, it has been reported that enslaving force in non-instructed fingers starts to increase at the same instance as force produced by the instructed finger [2]. No range of independent force production appears to be present. Furthermore, enslaving forces in multi-finger tasks were lower than in single-finger tasks, whereas in the movement tasks in our study, enslaving movement increased for the ring and little finger in multi-finger tasks. In force tasks, minimal finger movement is involved. The effects of mechanical coupling mediated by muscle-tendon lengthening are, therefore, likely minimal. Whereas Zatsiorsky et al. (2000) concluded that neural interaction is the main mechanism of force enslaving, the presence of the range of independent movement in our results indicate that, in contrast to force tasks, mechanical connections between muscle heads and tendons certainly play a crucial role during finger movements.

### Applications

Our data show that some degree of independence is present for small finger movements. Also, a quantitative description of the whole finger trajectory, up to full flexion with large length changes of muscle-tendon units, adds to the current literature on mechanical and neural factors on finger independency [10]. The present study therefore does provide insight into the role of finger independency for different type of tasks and different pathologies.

The extent of finger independency and selective control of individual fingers is of high relevance for musicians such as piano players [20, 21, 31]. The variable and asymmetric range of independent movement could play a large role in the small movements performed in piano playing. It could explain individual differences found in non-striking fingers adjacent to the striking finger between piano players [20]. Furthermore, evaluation of the *δ* individuation index and the range of independent movement might give further insight into changes of independent finger control by musical training [21].

Musicians and others performing precise and repetitive finger motor tasks are of risk to develop focal task specific dystonia [22, 23], which is characterized by a loss of independent movement. Focal task specific dystonia has been associated with impaired surround inhibition. Surround inhibition is a neural mechanism in which task relevant muscles are selectively activated, whereas neighboring muscles are inactivated [22, 36]. The enslaving and independency outcome measures used in our study could be helpful to predict or monitor the progression of such a disorder. Furthermore, it might increase our understanding of changes in finger motor control due to a stroke [37, 38] or aging [39]. After stroke, enslaving movement appears to be increased [37]. With aging, the structural, biochemical and functional characteristics of a skeletal muscle’s extracellular matrix, as well as the mechanical properties of tendons change [39, 40]. This affects the performance of finger manipulation tasks in these populations. Further research on the contribution of mechanical and neural factors in these populations is necessary in order to assess progression and to guide treatment.

### Methodological issues

A factor that could influence enslaving and the range of independent movement is the activity of antagonistic muscles, such as the extensor digitorum muscle [3]. In our study, at the beginning of the flexion movement, the finger extensors might still be active since 0 degrees of finger joint flexion/extension (starting position in our experiment) is not the anatomical resting posture [19, 41]. Furthermore, the extensor muscles might be active to minimize the enslaving movement, although subjects were instructed not to resist any natural movement in non-instructed fingers. This calls for assessing muscle activation in both agonist and antagonist muscles in further studies [9].

In the current study we used miniature inertial and magnetic sensors (the PowerGlove system) that enable accurate measurement of 3D joint angles [24, 25]. Previous studies used less accurate instrumented gloves such as the CyberGlove or goniometers [1, 17]. The PowerGlove sensors did not limit the natural movement of the fingers and all three finger joints of all fingers could be measured.

To express the finger enslaving, the joint angles of MCP, PIP and DIP were summed into the ΣROM. This parameter reflects the total trajectory of the fingertip and was considered to be a representative measure for the whole finger movement. However, a direct comparison in ROM of separate joints between studies could not be made.

The threshold applied to detect the start of movement in the non-instructed fingers is important to interpret the range of independent movement. We used a threshold of 5 degrees for the sum of all three joint angles, which means only 1 or 2 degrees in each joint. This threshold was based on studies investigating finger proprioception reporting that finger displacements below 2.5–5 degrees cannot be sensed [26, 27]. With respect to the total ΣROM of the instructed finger, this threshold represents about 3–4% of the whole movement range. Increasing the threshold will lead to higher estimates of range of independent movement, resulting in false positives (i.e. a range of independent movement is determined while it is not really present). In our opinion, the strict threshold we used resulted in valid approximations of such ranges.

The full finger flexion was performed at only one frequency (0.5Hz). It has been shown that finger movement performed at higher speed results in less independency as expressed by lower individuation index values [4]. Future studies might focus on a variety of frequencies and the measurement of EMG to gain further insight in the neurological aspects of the enslaving effect and the range of independent movement.

## Conclusions

We conclude that while no finger can move independently through the full range of finger flexion, some degree of independence is present for smaller finger movements. This range of independent movement is asymmetric and variable. These results, quantified with a precise measurement device using inertial sensing, provide insight into the role of finger independency for different types of tasks and populations.

## Supporting Information

S1 File Data_perSubject_perTask_perFinger. (XLSX)

## References

[1] Lang CE, Schieber MH. Human finger independence: Limitations due to passive mechanical coupling versus active neuromuscular control. Journal of Neurophysiology. 2004;92(5):2802–10. 10.1152/jn.00480.2004 15212429

[2] Zatsiorsky VM, Li ZM, Latash ML. Enslaving effects in multi-finger force production. Exp Brain Res. 2000;131(2):187–95. 10766271 10.1007/s002219900261

[3] Sanei K, Keir PJ. Independence and control of the fingers depend on direction and contraction mode. Human Movement Science. 2013;32(3):457–71. 10.1016/j.humov.2013.01.004 23643494

[4] Hager-Ross C, Schieber MH. Quantifying the independence of human finger movements: Comparisons of digits, hands, and movement frequencies. Journal of Neuroscience. 2000;20(22):8542–50. 11069962 10.1523/JNEUROSCI.20-22-08542.2000 PMC6773164

[5] Keen DA, Fuglevand AJ. Role of intertendinous connections in distribution of force in the human extensor digitorum muscle. Muscle & Nerve. 2003;28(5):614–22. 14571465 10.1002/mus.10481

[6] Maas H, Sandercock TG. Force Transmission between Synergistic Skeletal Muscles through Connective Tissue Linkages. Journal of Biomedicine and Biotechnology. 2010. 10.1155/2010/575672 PMC2853902 20396618

[7] Maas H, Jaspers RT, Baan GC, Huijing PA. Myofascial force transmission between a single muscle head and adjacent tissues: length effects of head III of rat EDL. Journal of Applied Physiology. 2003;95(5):2004–13. 10.1152/japplphysiol.00220.2003 12844495

[8] Maas H, Huijing PA. Myofascial force transmission in dynamic muscle conditions: effects of dynamic shortening of a single head of multi-tendoned rat extensor digitorum longus muscle. Eur J Appl Physiol. 2005;94(5–6):584–92. 10.1007/s00421-005-1367-7 15952026

[9] Leijnse JNAL, Campbell-Kyureghyan NH, Spektor D, Quesada PM. Assessment of Individual Finger Muscle Activity in the Extensor Digitorum Communis by Surface EMG. Journal of Neurophysiology. 2008;100(6):3225–35. 10.1152/jn.90570.2008 18650306

[10] van Duinen H, Gandevia SC. Constraints for control of the human hand. Journal of Physiology-London. 2011;589(23):5583–93. 10.1113/jphysiol.2011.217810 PMC3249034 21986205

[11] Schieber MH, Hibbard LS. How Somatotopic Is the Motor Cortex Hand Area. Science. 1993;261(5120):489–92. 8332915 10.1126/science.8332915

[12] Schieber MH, Santello M. Hand function: peripheral and central constraints on performance. Journal of Applied Physiology. 2004;96(6):2293–300. 10.1152/japplphysiol.01063.2003 15133016

[13] Keen DA, Fuglevand AJ. Common input to motor neurons innervating the same and different compartments of the human extensor digitorum muscle. Journal of Neurophysiology. 2004;91(1):57–62. 10.1152/jn.00650.2003 12968013

[14] Shinoda Y, Yokota J, Futami T. Divergent projection of individual corticospinal axons to motoneurons of multiple muscles in the monkey. Neurosci Lett. 1981;23(1):7–12. 6164967 10.1016/0304-3940(81)90182-8

[15] Ejaz N, Hamada M, Diedrichsen J. Hand use predicts the structure of representations in sensorimotor cortex. Nat Neurosci. 2015;18(7):1034–40. 10.1038/nn.4038 26030847

[16] Kim SW, Shim JK, Zatsiorsky VM, Latash ML. Finger inter-dependence: Linking the kinetic and kinematic variables. Human Movement Science. 2008;27(3):408–22. 10.1016/j.humov.2007.08.005 18255182 PMC2481561

[17] Li ZM, Dun SC, Harkness DA, Brininger TL. Motion enslaving among multiple fingers of the human hand. Motor Control. 2004;8(1):1–15. 14973334 10.1123/mcj.8.1.1

[18] An KN, Ueba Y, Chao EY, Cooney WP, Linscheid RL. Tendon Excursion and Moment Arm of Index Finger Muscles. Journal of Biomechanics. 1983;16(6):419–25. 6619158 10.1016/0021-9290(83)90074-x

[19] Li ZM, Zatsiorsky VM, Latash ML. Contribution of the extrinsic and intrinsic hand muscles to the moments in finger joints. Clinical Biomechanics. 2000;15(3):203–11. 10656982 10.1016/s0268-0033(99)00058-3

[20] Furuya S, Flanders M, Soechting JF. Hand kinematics of piano playing. Journal of Neurophysiology. 2011;106(6):2849–64. 10.1152/jn.00378.2011 21880938 PMC3234081

[21] Furuya S, Nakamura A, Nagata N. Acquisition of individuated finger movements through musical practice. Neuroscience. 2014;275:444–54. 10.1016/j.neuroscience.2014.06.031 24973654

[22] Furuya S, Hanakawa T. The curse of motor expertise: Use-dependent focal dystonia as a manifestation of maladaptive changes in body representation. Neuroscience Research. 2016;104:112–9. 10.1016/j.neures.2015.12.001 26689332

[23] Rosenkranz K, Butler K, Williamon A, Rothwell JC. Regaining Motor Control in Musician's Dystonia by Restoring Sensorimotor Organization. Journal of Neuroscience. 2009;29(46):14627–36. 10.1523/JNEUROSCI.2094-09.2009 19923295 PMC2998172

[24] Kortier HG, Sluiter VI, Roetenberg D, Veltink PH. Assessment of hand kinematics using inertial and magnetic sensors. Journal of Neuroengineering and Rehabilitation. 2014;11:70–83. 10.1186/1743-0003-11-70 24746123 PMC4019393

[25] van den Noort JC, Kortier HG, van Beek N, Veeger HE, Veltink PH. Measuring 3D hand and finger kinematics—a comparison between inertial sensing and an opto-electronic system. PloS One. 2016;11(11):16. Epub Nov 3, 2016. 10.1371/journal.pone.0164889 PMC5094774 27812139

[26] Clark FJ, Burgess RC, Chapin JW, Lipscomb WT. Role of intramuscular receptors in the awareness of limb position. J Neurophysiol. 1985;54(6):1529–40. 4087047 10.1152/jn.1985.54.6.1529

[27] Wycherley AS, Helliwell PS, Bird HA. A novel device for the measurement of proprioception in the hand. Rheumatology (Oxford). 2005;44(5):638–41. 15728416 10.1093/rheumatology/keh568

[28] Zatsiorsky VM, Li ZM, Latash ML. Coordinated force production in multi-finger tasks: finger interaction and neural network modeling. Biol Cybern. 1998;79(2):139–50. 10.1007/s004220050466 9791934

[29] Marieb EN. The Muscular System Human Anatomy & Physiology. 5th ed United States of America: Addison Wesley Longman, Inc.; 2001 p. 357.

[30] von Schroeder HP, Botte MJ. Anatomy of the extensor tendons of the fingers: variations and multiplicity. J Hand Surg Am. 1995;20(1):27–34. 10.1016/S0363-5023(05)80053-X 7722260

[31] Leijnse JNAL. Measuring force transfers in the deep flexors of the musician's hand: Theoretical analysis, clinical examples. Journal of Biomechanics. 1997;30(9):873–82. 9302609 10.1016/s0021-9290(97)00045-6

[32] Merzenich MM, Nelson RJ, Stryker MP, Cynader MS, Schoppmann A, Zook JM. Somatosensory cortical map changes following digit amputation in adult monkeys. J Comp Neurol. 1984;224(4):591–605. 10.1002/cne.902240408 6725633

[33] Schieber MH. Motor cortex and the distributed anatomy of finger movements. Adv Exp Med Biol. 2002;508:411–6. 12171137 10.1007/978-1-4615-0713-0_46

[34] Danion F, Latash ML, Li S. Finger interactions studied with transcranial magnetic stimulation during multi-finger force production tasks. Clin Neurophysiol. 2003;114(8):1445–55. 12888027 10.1016/s1388-2457(03)00105-6

[35] van Beek N, Stegeman D, van den Noort JC, Veeger HE, Maas H. Neuromuscular control of extrinsic flexors and extensors during single finger movements In proceedings of XXI congres of International Society of Electrophysiology and Kinesiology; 2016 Chigaco, United States 2016.

[36] Sohn YH, Hallett M. Surround inhibition in human motor system. Experimental Brain Research. 2004;158(4):397–404. 10.1007/s00221-004-1909-y 15146307

[37] Schieber MH, Lang CE, Reilly KT, McNulty P, Sirigu A. Selective activation of human finger muscles after stroke or amputation. Adv Exp Med Biol. 2009;629:559–75. 10.1007/978-0-387-77064-2_30 19227521 PMC2712614

[38] Raghavan P, Santello M, Gordon AM, Krakauer JW. Compensatory Motor Control After Stroke: An Alternative Joint Strategy for Object-Dependent Shaping of Hand Posture. Journal of Neurophysiology. 2010;103(6):3034–43. 10.1152/jn.00936.2009 20457866 PMC2888236

[39] Kragstrup TW, Kjaer M, Mackey AL. Structural, biochemical, cellular, and functional changes in skeletal muscle extracellular matrix with aging. Scandinavian Journal of Medicine & Science in Sports. 2011;21(6):749–57. 22092924 10.1111/j.1600-0838.2011.01377.x

[40] Latash ML, Shim JK, Shinohara M, Zatsiorsky VM. Changes in finger coordination and hand function with advanced age. Motor control and learning 2006 p. 141–59.

[41] Napier JR. Hands: revised by R. Tuttle. Princeton: Princeton University Press; 1993 1993.

